# Supplementary material for: Mechanistic insights into the active site and allosteric communication pathways in human nonmuscle myosin-2C
Source: eLife. 2017 Dec 19;6:e32742. doi: 10.7554/eLife.32742 (PMC5749951; doi:10.7554/eLife.32742)
Supplement: Supplementary file 1. [file elife-32742-supp1.docx]

**Supplementary file 1**

**Dihedral angles of switch-1 and lever arm residues in crystal structures of NM2C and smooth muscle myosin-2 (PDB entry 1BR2).**

| ***Hs* NM2C** | | | ***Gg* smooth muscle myosin-2** | | |
| --- | --- | --- | --- | --- | --- |
| Residue | φ | ψ | Residue | φ | ψ |
| *Switch-1* |  |  |  |  |  |
| N256 | -152.2 | 125.4 | N242 | -148.5 | 139.7 |
| D257 | -66.7 | -15.6 | D243 | -78.1 | -32.9 |
| N258 | -135.6 | 51.3 | N244 | -116.9 | 55.9 |
| S259 | -71.5 | 128.2 | S245 | -81.2 | 127.9 |
| S260 | -78.1 | 122.8 | S246 | -71.1 | 119.6 |
| R261 | -104.7 | 29.2 | R247 | -102.5 | 35.4 |
| F262 | -161.6 | 156.6 | F248 | -168.3 | 141.5 |
| G263 | -98.8 | 156.5 | G249 | -78.8 | 165.2 |
| K264 | -148.4 | 141.1 | K250 | -159.4 | 129.9 |
| *Lever arm* |  |  |  |  |  |
| F786 | -95.4 | 133.3 | F775 | -96.73 | 131.6 |
| F787 | -124.1 | 154.2 | F776 | -113.62 | 151.8 |
| R788 | -72.2 | 159.4 | R777 | -73.08 | 164.5 |
| A789 | -55.2 | 139.0 | T778 | -54.84 | 137.1 |
| G790 | 82.4 | -3.7 | G779 | 64.23 | 15.6 |
| V791 | -72.2 | -41.3 | V780 | -81.43 | -54.4 |
| L792 | -72.2 | -41.2 | L781 | -58.17 | -35.6 |
| A793 | -54.7 | -36.7 | A782 | -48.66 | -63.9 |
| Q794 | -78.2 | -30.3 | H783 | -53.48 | -37.6 |
| L795 | -75.2 | -32.8 | L784 | -61.41 | -42.1 |
| E796 | -67.5 | -31.7 | E785 | -63.52 | -52.9 |
| E797 | -79.4 | -32.2 | E786 | -52.85 | -42.9 |
| E798 | -74.2 | -36.6 | E787 | -66.4 | -36.1 |
